# Toward an Improved Air Pollution Warning System in Quebec

**DOI:** 10.3390/ijerph16122095

**Published:** 2019-06-13

**Authors:** Pierre Masselot, Fateh Chebana, Éric Lavigne, Céline Campagna, Pierre Gosselin, Taha B.M.J. Ouarda

**Affiliations:** 1 Institut National de la Recherche Scientifique, Centre Eau-Terre-Environnement, 490, rue de la Couronne, Québec, QC G1K 9A9, Canada; fateh.chebana@ete.inrs.ca (F.C.); celine.campagna@inspq.qc.ca (C.C.); pierre.gosselin@inspq.qc.ca (P.G.); taha.ouarda@ete.inrs.ca (T.B.M.J.O.); 2 School of Epidemiology and Public Health, University of Ottawa, 600 Peter Morand Crescent, Ottawa, ON K1G 5Z3, Canada; eric.lavigne@canada.ca; 3 Air health Science Division, Health Canada, 269 Laurier Ave West, Ottawa, ON K1A 0K9, Canada; 4 Institut National de Santé Publique du Québec, 945 Avenue Wolfe, Québec, QC G1V 5B3, Canada; 5 Ouranos, 550 Rue Sherbrooke Ouest, Montréal, QC H3A 1B9, Canada

**Keywords:** warning system, air pollution, respiratory diseases, cardiovascular diseases, mortality, threshold

## Abstract

The nature of pollutants involved in smog episodes can vary significantly in various cities and contexts and will impact local populations differently due to actual exposure and pre-existing sensitivities for cardiovascular or respiratory diseases. While regulated standards and guidance remain important, it is relevant for cities to have local warning systems related to air pollution. The present paper proposes indicators and thresholds for an air pollution warning system in the metropolitan areas of Montreal and Quebec City (Canada). It takes into account past and current local health impacts to launch its public health warnings for short-term episodes. This warning system considers fine particulate matter (PM_2.5_) as well as the combined oxidant capacity of ozone and nitrogen dioxide (O_x_) as environmental exposures. The methodology used to determine indicators and thresholds consists in identifying extreme excess mortality episodes in the data and then choosing the indicators and thresholds to optimize the detection of these episodes. The thresholds found for the summer were 31 μg/m^3^ for PM_2.5_ and 43 ppb for O_x_ in Montreal, and 32 μg/m^3^ and 23 ppb in Quebec City. In winter, thresholds found were 25 μg/m^3^ and 26 ppb in Montreal, and 33 μg/m^3^ and 21 ppb in Quebec City. These results are in line with different guidelines existing concerning air quality, but more adapted to the cities examined. In addition, a sensitivity analysis is conducted which suggests that O_x_ is more determinant than PM_2.5_ in detecting excess mortality episodes.

## 1. Introduction

Air pollution is a major public health issue with number of epidemiological studies reporting impact of diverse air pollutant on both mortality and morbidity [1]. In particular, respiratory and cardiovascular diseases are particularly impacted by air pollution [2,3]. The most studied pollutants are fine particulate matters (PM_2.5_) [4,5], ozone (O_3_) [6], and nitrogen dioxide (NO_2_) [7]. 

PM_2.5_ is an important cause of respiratory and cardiovascular diseases, by penetrating into the respiratory system [8]. Emissions of PM_2.5_ are mainly caused by fossil fuel combustion, but can also originate from wildfire smoke [9,10]. O_3_ originates from chemical reactions in the atmosphere which are catalyzed by solar radiation, and is thus more prominent during summer. It may exacerbate respiratory diseases such as asthma [11,12]. NO_2_ is generated by combustion with a prominent source being motorized vehicles, especially in a place like the province of Quebec where electric power generation comes mainly from hydraulic sources, including home heating. 

Adverse effects of air pollution led many public health authorities to implement early-warning systems [13]. Such systems have been implemented in many developed countries such as the UK, United States, and France. Intensive research is also currently conducted in China to improve the performances of early-warning systems [14,15]. In Quebec, air quality indicators and threshold guidelines are already used both at the Canadian [16,17] and the provincial [18] level. 

Current early-warning systems usually involve the monitoring of an air quality index (AQI) and the use of a predefined threshold or scale (i.e., several thresholds) to inform the population of potential risks [13]. The focus of research on early warning systems usually concerns the improvement of AQI modelling and forecasting [19], which is nowadays accurate. However, much less attention is given to the choice of thresholds, which are mainly based on overall reviews of risk assessment [20] and are thus largely related to risk management. The main weakness of early-warning systems is thus that the thresholds do not necessarily accurately reflect the impact of AQIs or air pollutants on public health. Consequently, the thresholds and resulting guidelines are very similar from country to country and poorly reflect the local population characteristics or the actual mix of pollutants. 

Recent reviews of air pollution and health impacts show wide worldwide variation of PM_2.5_ concentrations, with high variability in effect estimates across cities, up to an order of magnitude [21]. They also show, in the Canadian context, adverse effects of outdoor air pollution at concentrations that were below existing North American standards for children. Moreover, heterogeneous effects of air pollutants were found according to city, sex, socioeconomic status, and seasonality [22]. There seems to be specific impacts on the elderly but few studies have specifically addressed this context [23]. In addition, the health impact of each of these pollutants may vary according to the season, by interacting with temperature [24]. This diversity of the effects stresses the need to adapt local air pollution thresholds and guidelines to the population’s distinctive characteristics, including health status. 

Following this need, the present study proposes an air pollution-health warning system (APHWS) in the two largest cities of the province of Quebec: Montreal and Quebec City. This APHWS seeks to adapt indicators and thresholds to each city population, by not being based on general guidelines but on a data-based evaluation of the air pollution levels that lead to increased risks on health, and in particular mortality. Note that few studies sought to propose air pollution guidelines based on health, such as Cairncross et al. [25], which weighs the pollutants in the AQI based on estimated relative risks (RR) on health. Islam et al. [26] set a threshold for PM_10_ in the context of a global evaluation of climate warning thresholds for the Greater London. However, these ideas are based on the interpretation of a dose–lag–response relationship and not on the direct estimation of thresholds. 

In the present study, we consider a methodology that allows estimating thresholds and indicators on historical data of air pollution and health endpoints. In particular, the methodology relates past records of important excess mortality events to high air pollution levels. This allows an accurate assessment of thresholds above which air pollution is considered an important threat for the population’s health. The considered method is adapted from a previously proposed one in the context of heat-health warning systems (HHWS) [27]. In the heat context, such a method allowed an efficient prediction of excess mortality events [28] and the resulting HHWS effectively helped in reducing heat-related mortality during summer [29]. Thus, we expect similar benefits from its adaptation to the air pollution context.

## 2. Materials and Methods

### 2.1. Data

The data used in this study are those of the two main metropolitan areas of the province of Quebec: Quebec City and Montreal. As exposure indicators, daily maximum values of fine particulate matter (PM_2.5_), ozone (O_3_), and nitrogen dioxide (NO_2_) are collected by the National Air Pollution Surveillance (NAPS) program of Environment and Climate Change Canada (ECCC). These three pollutants are chosen because they usually stand out in the impact of air pollution on health [1,17]. The time series used corresponds to the spatial mean of the measurement stations inside the two cities’ metropolitan areas. 

The proposed APHWS seeks to prevent extreme excess mortality events, which are rare by nature. It thus seems unlikely that these events occur while the three considered pollutants simultaneously show elevated values. This is notably due to the so-called curse of dimensionality [30]. Therefore, instead of directly using O_3_ and NO_2_, we compute their combined oxidant capacity (O_x_) as $O_{x}=\left(2.075\times O_{3}+1.07\times {\mathrm{NO}}_{2}\right)/3.145$ [31]. Indeed, it has been shown that considering O_x_ is equivalent to considering both O_3_ and NO_2_ [32,33].

A preliminary analysis of air-pollution-related mortality also includes temperature and relative humidity. They are collected from the DayMet database maintained by the National Aeronautics and Space Administration (NASA). DayMet provides reanalyzed values for several meteorological variables on a spatial grid with a resolution of 1 km. Grid points inside the ecumene of the metropolitan areas are averaged to obtain a single series for each city. Note that DayMet does not directly provide relative humidity but vapor pressure. The former is thus calculated from the latter using standard formulas [34].

The health impact of the APHWS is based on mortality series for which at least one of the causes (main or secondary) inherits from either cardiovascular (codes I10–I13, I15, I20–I25, I50 of the ICD10) or respiratory diseases (codes J00–J99 of the ICD10). These systems are the most vulnerable to air pollution and jointly considering them allows obtaining a large number of events [6]. These data were provided by the Institut national de santé publique du Québec (INSPQ).

Exposure and health data are jointly available from 1998 to 2014. However, the years 1998 and 1999 are omitted from the dataset to avoid issues related to the ICD change (from 9 to 10) in 2000, which results in a non-negligible breakpoint in the respiratory mortality series [35]. This leaves a total of 15 years of data. Since the association between air pollution and mortality vary with temperature, the APHWS is thus separated into two seasonal components: Summer (May to September) and winter (October to April). Thus, the final series lengths are $n_{s}=2295$ days and $n_{w}=3184$ days, respectively. Table 1 shows the mean daily values of each variable considered in the present study for Montreal and Quebec City and for both summer and winter.

### 2.2. Statistical Methodology

The considered statistical methodology was initially developed for France’s HHWS [36] and thereafter generalized and adapted to the province of Quebec [27]. The goal of this methodology is to determine two couples of indicators $Z_{t}^{\left(j\right)}$ and thresholds $s^{\left(j\right)}$ on air pollutants (where j stands for PM_2.5_ or O_x_) such that when both $Z_{t}^{\left(j\right)}>s^{\left(j\right)}$, day t is considered as an alarm. They are determined through the four following steps, which are then described with more details below:

1. Choose maximum lags for air pollution indicators;
2. Compute an excess mortality (EM) series from the mortality data;
3. Determine extreme EM episodes as targets of the APHWS;
4. Choose the best indicators–thresholds combination.

Note that each of these steps is performed once for each seasonal APHWS and for each city.

#### 2.2.1. Choose Maximum Lags for Air Pollution Indicators

In the present APHWS, we consider indicators $Z_{t}^{\left(j\right)}$ as weighted means of past daily pollutant values, i.e.,:

(1) $$Z_{t}^{\left(j\right)}=\overset{L}{\underset{l=0}{\sum }}\alpha_{l}X_{t-l}^{\left(j\right)}$$

where $X_{t}^{\left(j\right)}$ are daily values of air pollutant j (PM_2.5_ or O_x_) and $\alpha_{l}$ are weights to be optimized. We constrain the weights such that $\sum_{l}\alpha_{l}=1$ and $\alpha_{0}\geq \;\alpha_{1}\geq \ldots \geq \alpha_{L}$. The latter constraint accounts that the APHWS will be running on forecast data (0 to 3 days) and therefore ensures that the importance of each daily forecast in the indicator decreases with the horizon.

The purpose of the first step is to determine the value of the maximum lag L in Equation (1) through a preliminary analysis. It can be chosen by fitting a distributed lag nonlinear model (DLNM) [37]. A DLNM accounts for both measured and unmeasured confounders (by opposition to more common methods such as the cross-correlation function) and yields a lag–response relationship to visually choose L. Here, quasi-Poisson DLNMs are fitted with penalized splines on the variable dimension [38] and natural splines on the lag dimension. The latter includes lags from 0 to 5 days, with interior knots at lags 1 and 2 to account for the very short-term acute effect of air pollution indicators on mortality [4]. Smooth spline components of time are used to account for unmeasured confounder, one for the day of season with four degree of freedoms and one for the long-term trend with one degree of freedom per decade as in, e.g., Gasparrini et al. [39]. Weather confounding is also accounted for, by including temperature and relative humidity both averaged over the three previous days. Temperature is included in a natural spline with three degrees of freedom to model the U-shaped relationship, and relative humidity is included in a linear fashion [32,40].

#### 2.2.2. Compute an Excess Mortality Series

The excess mortality (EM) series represents excess death from a baseline of expected mortality. It is computed as:

(2) $$EM_{t}=\frac{Y_{t}-B_{t}}{B_{t}}\times 100$$

where $Y_{t}$ is the observed mortality series and $B_{t}$ is the mortality baseline, i.e., the expected mortality without influence of the exposure of interest. $B_{t}$ is estimated in a standard manner, i.e., through natural cubic splines with eight degrees of freedom per year. It accounts for seasonal variations and the long-term trend of mortality.

#### 2.2.3. Determine Significant Excess Mortality Episodes

The role of this step is to choose the magnitude of EM (as defined in Equation (2)) to be detected by the APHWS. This is translated by choosing a preliminary threshold $s_{OM}$ on EM and considering that all days for which EM exceeds this threshold are extremes. This threshold can vary by location. It ought to be a compromise between a sufficient number of EM extremes and important enough extremes which are not due to statistical fluctuations.

Since extreme EM days tend to occur in clusters [41], we consider any cluster as an EM episode. Episodes are hereby defined as a cluster of EM extremes separated by less than three days from each other, with the addition of the three days preceding and the three days following the cluster of extremes. In practice, we consider that an EM episode is successfully detected if at least one of its days is detected. It allows accounting for the temporal dependence between extreme days.

Finally, an additional constraint is added to stipulate that the same day maximum PM_2.5_ exceeds the value of 25 μg/m^3^. This ensures that the EM episode is related to high levels of air pollution and not only to potentially confounding events, such as heat waves or cold spells. The preliminary value of 25 μg/m^3^ is based on the regulatory criteria advised by the World Health Organisation [19] as an acceptable prior.

#### 2.2.4. Choose the Best Combination of Indicators–Thresholds

In this final step of the methodology, the optimal indicator weightings $\alpha_{l}$ (in Equation (1)) and threshold s are chosen. Different weighting and threshold combinations are tested and, for each of them, resulting alarm days are compared to the actual EM episodes determined in step 3. For given weights (constrained as explained in Section 2.2.1) and thresholds, alarm days t are determined such that $Z_{t}^{\left(j\right)}>s^{\left(j\right)}$ for each indicator. It includes all days which would have been set as alerts considering chosen weights and thresholds.

The relevancy of weightings and threshold can be evaluated by comparing the obtained alarms with the episodes extracted in step 3 (Section 2.2.3). Two criteria are considered: (1) Sensitivity, which is the proportion of actual EM episodes (determined in the previous step) that are correctly detected by the APHWS and (2) the number of false alarms (FA) which are false positive episodes. Note that, even though a specificity score is usually used in binary classification [36], in the present case, the very low number of extremes lead to specificity scores always very close to 1. Specificity is thus not very informative and the number of FA is preferred to evaluate the accuracy of an APHWS. The chosen weightings and thresholds should represent a trade-off between high sensitivity and low number of false alarms. We nonetheless attribute a larger importance to sensitivity since the APHWS is intended for public health authorities which decide to actually perform actions whenever the APHWS gives them a signal.

## 3. Results

The focus of the present section is on the results of Montreal to show the process of choosing thresholds and indicators for the APHWS. However, figures and tables showing results for Quebec City can be found in the Supplementary Materials (Figures S1–S3 as well as Tables S1 and S2).

### 3.1. Results for Montreal’s APHWS

#### 3.1.1. Choice of Lags

**Figure 1:**
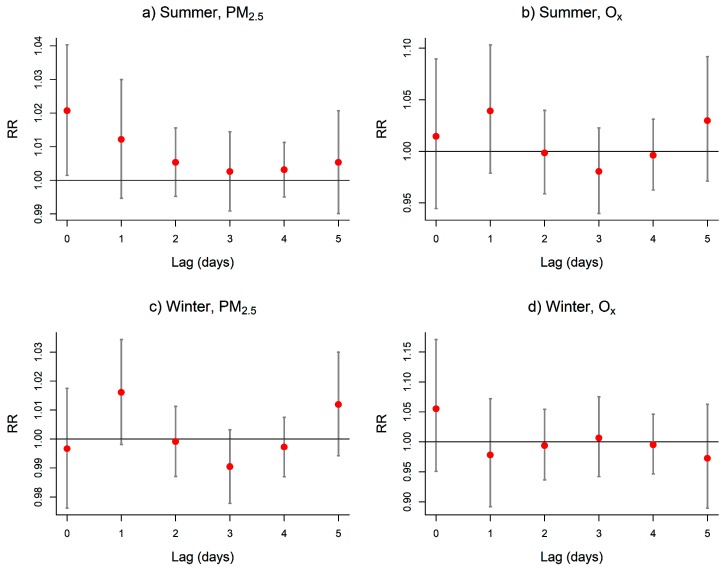
Lag–response relationship between mortality and (**a**,**c**) PM_2.5_ and (**b**,**d**) O_x_ in Montreal. These curves correspond to the slice of the distributed lag nonlinear model (DLNM) surface at values ${\mathrm{PM}}_{2.5}=25\;\mu g/m^{3}$ and $O_{x}=50\;\mathrm{ppb}$. Gray bars indicate 95% confidence intervals. RR = relative risk.

Figure 1 shows the lag–response relationships between the pollutants and mortality at the values ${\mathrm{PM}}_{2.5}=25\;\mu g/m^{3}$ and $O_{x}=50\;\mathrm{ppb}$. These levels correspond to previous guidelines on adverse effects of these indicators on health. Note that the whole DLNM surface can be found in the Supplementary Material (Figure S4) but brings little additional information since the dose–response relationship is quasilinear. In summer for PM_2.5_, Figure 1a suggests a relative risk (RR) which significantly differs from 1 on the same day only (lag 0), but also in a non-negligible way at lag 1. The RR then rapidly decreases toward 1 when the lag increases. We chose L=1 for the PM_2.5_ indicator in the summer APHWS to avoid underestimating its impact on mortality [4]. Figure 1b shows that the lag–response relationship with O_x_ is the largest at lag 1 and is very close to RR = 1 for lags larger than 1, with the exception of lag 5 which could be a boundary effect of the model. In summer, the chosen maximum lag for the O_x_ indicator is then also L=1.

In winter, Figure 1c shows that the RR of PM_2.5_ is at his highest at lag 1 and then RR immediately shrinks toward 1. Although the RR of O_x_ is close to 1 at all lags, we nonetheless chose an indicator with lag 1 for the O_x_ indicator in winter since prior research showed that it could also have an impact at lag 1 [32]. To summarize, all indicators considered in the following are two-day weighted means of pollutants, i.e., considering lags 0 and 1.

#### 3.1.2. Excess Mortality Episodes

Step 2 of the procedure is to compute EM using Equation (2). Summary statistics of estimated EM are shown in Table 2. It shows that the baseline estimates well the expected mortality since EM series are centered at 0%. EM distribution is similar between summer and winter with slightly lower variations in winter than in summer. This is especially true at extreme values since the maximum observed EM in summer is 110.4% while the maximum in winter is 58.8%. These maxima correspond respectively to the 8 July 2010 which occurred during a major heat wave [42], and to the 9 November 2013 which does not seem to correspond to a particular event. 

**Figure 2:**
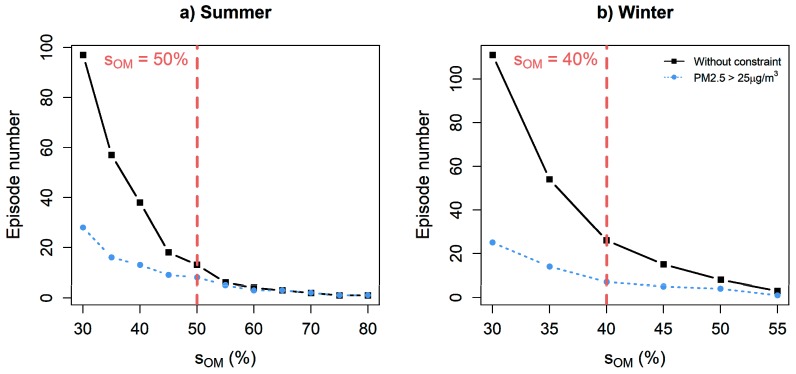
Number of EM episodes according to the chosen preliminary EM threshold $s_{OM}$.

Step 3 consists of choosing the preliminary threshold $s_{OM}$ indicating the minimum EM above which a day is considered as an EM extreme. Figure 2 indicates the number of episodes found in the data according to different $s_{OM}$ values for summer and winter. Recall that an episode may contain several exceeding days if they are closer than three days from each other. Figure 2a shows that, in summer, for $s_{OM}<50\%$, the number of episodes associated with important PM_2.5_ is much lower than the sole number of episodes (without constraint). However, above 50%, the majority of the episodes are associated with an amount of PM_2.5_ larger than 25 μg/m^3^. The value $s_{OM}=50\%$ is thus chosen for summer as the preliminary threshold to determine significant EM episodes. 

In winter, Figure 2b indicates that a large number of EM episodes are not linked to important amount of air pollution. The blue curve showing the number of episodes with PM_2.5_ larger than 25 μg/m^3^ rapidly decreases until $s_{OM}=40\%$. We therefore chose $s_{OM}=40\%$ for winter, which is slightly lower than in summer, consistently with the summary statistics of EM shown in Table 2.

**Figure 3:**
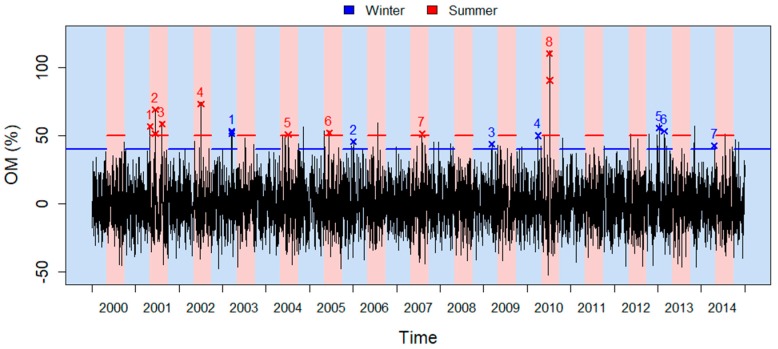
EM series with the identification of EM episodes. Crosses indicate extreme EM days (exceeding $s_{OM}$) and the number identifies episodes. Horizontal segments identify $s_{OM}$. Red areas identify summer and blue ones winter. Note that nonextreme days extending the episodes are not identified here for clarity purposes.

Figure 3 shows the episodes identified with the thresholds $s_{OM}=50\%$ in summer and $s_{OM}=40\%$ in winter. Although summer is hereby shorter than winter, overall, more episodes are detected in the former, i.e., eight episodes in summer and seven in winter. This may be due to the synergy between temperature and pollution for which a growing body of evidence exists [24,43]. Indeed, among the eight summer episodes, four of them (the 2nd, 3rd, 4th, and 8th) have been already detected in a heat wave context [44] and correspond to the highest EM values. For instance, the deadly heat wave of July 2010 is clearly identifiable as the 8th episode. Note also that the 4th episode corresponds to the important forest fires of July 2002 [45]. Supplementary details about the identified episodes are given in Tables S3 and S4 (Supplementary Materials).

#### 3.1.3. Final Indicators and Thresholds

The results for different indicator weightings $\alpha_{l}$ and thresholds s with associated scores are shown in Tables S5 and S6 for summer and winter respectively (Supplementary Materials) and the chosen ones are shown in Table 3. The rationale for choosing final indicators and thresholds is based on episodes: The chosen system should be a trade-off between an episode sensitivity close to one and a low number of episodes. 

In summer, the chosen system allocates the largest weight to same day PM_2.5_ and equal weights to the two days of O_x_. This is reversed in winter where the largest weight is the same day O_x_, while the two days of PM_2.5_ have equal weights. Note that this is consistent with the lag–response relationships shown in Figure 1. The thresholds found for summer are $s_{PM2.5}=31\;\mu g/m^{3}$ and $s_{Ox}=43\;\mathrm{ppb}$ and are much lower for winter at the values $s_{PM2.5}=25\;\mu g/m^{3}$ and $s_{Ox}=26\;\mathrm{ppb}$. 

The sensitivity and false alarms criteria show that the system has better performances in summer. Indeed, in summer, seven out of the eight episodes (87.5%) are detected by the system. As shown in Table S5 (Supplementary Materials), we preferred to miss one of the episodes to obtain a lower amount of false alarms. This missed episode is the 5th one (in July 2004) and is borderline since it shows a maximum EM of 51% while the preliminary threshold is at $s_{OM}=50\%$. Note that it corresponds to a particularly dry (relative humidity of 55%) and hot ($T_{max}=30\;{}^{\circ}C$) day. With the chosen system, only 1.5 false episodes are launched each year.

In winter, a system detecting only five out of the seven episodes is chosen, since it allows to launch half the number of false alarms than systems with better sensitivities (see Table S6 in Supplementary Materials). The two missed episodes are the 2nd and 5th one of January 2006 and 2013. Note that during the 5th episode, temperature is positive, which is unusual in January. The chosen system leads to an average of eight false alarms clustered in 3.7 false episodes per year. This system is thus less powerful than the summer one.

Table 3 also shows the final APHWS chosen in Quebec City. Indicators for both summer and winter are similar to indicators for winter in Montreal, i.e., equal weights for PM_2.5_ and larger weight on the same day for O_x_. The thresholds found for PM_2.5_ are similar to the summer system at Montreal at $s_{PM2.5}=32\;\mu g/m^{3}$ for summer and $s_{PM2.5}=33\;\mu g/m^{3}$ for winter, but the thresholds for O_x_ are much lower at $s_{Ox}=23$ and 21 ppb. The main difference is nonetheless in the performances, much lower in Quebec City than in Montreal. The sensitivities are overall lower and the number of false alarms higher. 

### 3.2. Sensitivity Analysis

**Figure 4:**
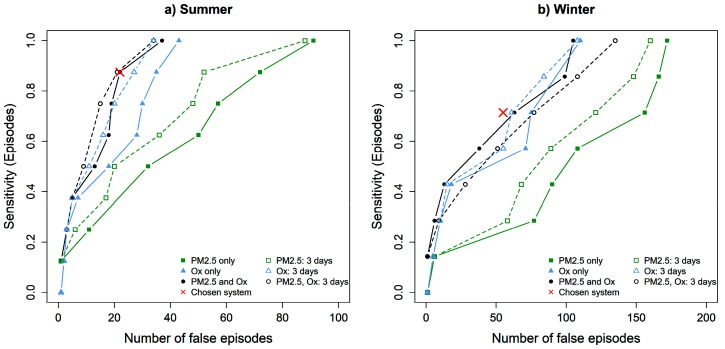
Receiver operating characteristic (ROC) curves for different variables used in the air pollution-health warning system (APHWS). For a type of APHWS, each point corresponds to the result of a particular weighting and threshold couple. The points reported here correspond to likely choices, i.e., to the best trade-offs between sensitivity and false alarms. Filled symbols correspond to APHWS with two-day indicators, and empty symbols to APHWS with three-day indicators. The red cross corresponds to the system of Table 3.

The variables and lags used to construct indicators are chosen based on prior knowledge of the relationship between atmospheric pollutants and mortality. However, it is of interest to evaluate the sensitivity of the APHWS to each variable and to the choice of lags. In this regard, Figure 4 shows the sensitivity versus the number of FA for different choices of variables and lags in Montreal. Variables are either PM_2.5_ alone, O_x_ alone, or both, as considered in Section 3.1. Lags are either L=1 as in Section 3.1 or L=2 to evaluate whether adding a day of lag would strengthen the APHWS. The closer to the upper left corner the curve, the better the APHWS. Note that this is similar to the well-known receiver operating characteristic (ROC) curves and thus we will refer to Figure 4 as ROC curves in the following.

Figure 4 shows that the APHWS is more sensitive to the presence of O_x_ than to the presence of PM_2.5_. Indeed, an APHWS with only O_x_ shows a ROC curve close to the APHWS with both variables, while an APHWS with only PM_2.5_ shows lower performances. This difference is more prominent in summer than winter, suggesting an important impact of O_x_ in summer. This result also strengthens the choice to consider O_x_ in the APHWS. 

Regarding the lag, Figure 4 shows that an APHWS using indicators with L=2 have overall ROC curves slightly closer to the upper left corner than their L=1 counterparts. The only exception is the system with two indicators in winter, showing a ROC curve above its L=2 counterpart. The difference is not important for APHWS with high thresholds (low sensitivity and false alarms) but grows as the sensitivity and number of false alarms increase. Therefore, the ROC curves suggest that considering L=2 could only marginally increase the APHWS’s strength. This legitimates the choice of L=1 by showing that increasing it would not really improve the APHWS 

## 4. Discussion

Many organizations have established air quality guidelines at different scales, such as the World Health Organization (WHO) at the global scale, Environment and Climate Change Canada (ECCC) for the country, the Ministère du Développement Durable de l’Environnement et de la Lutte contre les Changement Climatiques (MDDELCC) for the province of Quebec, and the Réseau de Surveillance de la Qualité de l’Air (RSQA) for the sole city of Montreal (about half the metropolitan area). One has to keep in mind that those guidelines are specific to the geographic level of management. For instance, decision makers should consider the Montreal value for the population of Montreal city, and the Province of Quebec values for the population in other locations of the province. Provincial values are mandatory, Canadian Ambient Air Quality Standards are recommendations based of health risk evaluation and technical feasibility of risk management, while the WHO guidelines are health-based values that do not consider management issues. The guidelines issued by these organizations are shown in Table 4 for comparison with our results. Note that no guidelines exist for O_x_, and the ones reported are (rough) estimations from guidelines for O_3_ and NO_2_ using the formula in Section 2.1 and should not be seen as standard.

The threshold values found for short-term exposures to PM_2.5_ $(31\;\mu g/m^{3}$ and $25\;\mu g/m^{3}$) are in the range of the different guidelines. The summer threshold is close to the one considered in the province of Quebec while the winter one is closer to the WHO recommendation. However, the thresholds found for O_x_ are much lower than the calculated guidelines. This may be explained by the different indicators which are based on very short-term exposure, while we hereby consider two-days means of maximum values. This could also be an indication that O_x_ have an impact at lower values than expected.

In the case of Quebec City, no guideline existed specifically for the city before the present study. However, Table 4 shows that the PM_2.5_ thresholds for Quebec City are similar to the Montreal’s ones and that the O_x_ thresholds are much lower than any values of the calculated guideline. Overall, the APHWS is less performant in Quebec City with more false alarms and lower sensitivity. This is due to the smaller population of Quebec City (800,000 versus around 4 million in Montreal, including metropolitan areas) which results in a lower amount of cardiovascular or respiratory mortality (a mean of three deaths per day). This makes setting an APHWS much more perilous since the response to an extreme AP exposure may be hidden in the day-to-day variations. This could explain the low values of $s_{Ox}$ for which there may not be enough mortality to estimate its impact on the population.

In summer, half of the determined episodes correspond to high temperature peaks (see Table S3) which could result in an overestimation of the response to smog episodes. This is consistent with the recent European study that showed a synergistic effect of hot temperature and air pollution on mortality [24]. However, note that in an operational context, we prefer risking overestimation of the mortality response rather than underestimation, especially since the APHWS is expected to be used jointly with the heat-health warning system. Finally, note that these episodes still correspond to important air pollution levels because of the preliminary threshold on PM_2.5_ (25 μg/m^3^). Thus, they cannot reasonably be discarded from the analysis. 

A number of limitations arise concerning the method considered for proposing the present APHWS. Indeed, the final indicators and threshold significantly depend on choices made in the preliminary steps of the method. For instance, extreme EM episodes linked to air pollution are determined using two predetermined and subjectively chosen thresholds: $s_{OM}$ and the preliminary threshold on PM_2.5_ (25 μg/m^3^). This may not be as adapted to air pollution as it is for heat waves, because of the seemingly linear mortality response to an air pollution exposure. This means that there are no values above which the relationship is stronger [47], in contrast with temperature for which minimum mortality intervals can be identified above which the risk is greatly increased [48]. In addition, although other variables, such as temperature, humidity and the day of season, can act as potential confounders, they are difficult to integrate to the methodology to choose thresholds and indicators.

Another limitation arises from the sole use of mortality as health issue as it is insufficient to represent the whole impact of smog episodes. Air pollution also impacts hospital admissions for instance [3]. However, hospital admissions do not allow for the detection of extreme impacts of smog the same way mortality does, as illustrated in Figure S5 (Supplementary Materials). Note that the APHWS could nonetheless be strengthened by also considering other data sources, such as the telephone service Info-Santé (811 in Quebec) or ambulance calls.

As a perspective, further methodological development is needed to estimate optimal indicators and threshold, accounting for the limitations exposed above. It could also be of interest to consider indicators similar to the WHO and ECCC guidelines, i.e., based on 1-h or 8-h maxima, rather than daily maxima. Indeed, the impact of O_x_ in particular seems to be very short-term.

Of course, the proposed modifications in thresholds are not intended for use by the general public that would get confused with several coexisting thresholds. Our intention is rather to provide the decision-maker with a more precise assessment of potential harm during air pollution peak episodes in a specific urban area. This could lead, for instance, to more vigorous pollutant reduction measures, such as free public transit or speed limitations, to decrease peak levels of pollution for a limited time period. Indeed, air pollution is by far the most important and expensive risk in Canada [49].

## 5. Conclusions

The present study offers a starting point to develop an air pollution-health warning system in the cities of Montreal and Quebec City of the province of Quebec, Canada. Here, we propose initial indicators and thresholds to be monitored. The analysis and methodology used to establish the APHWS are inspired by the previous work concerning heat waves. It consists in first determining a level of excess mortality to be detected by the APHWS and then choosing the best air pollutant indicators and thresholds in order to launch relevant alarms. Depending on the city and season, thresholds found range between 25 and 33 μg/m^3^ for PM_2.5_ and between 21 and 43 ppb for O_x_. Thresholds on PM_2.5_ are consistent with the guidelines of different organizations, but more focused on extreme events and thus more accurate. However, thresholds on O_x_ are lower than expected which could indicate that further research on its impact may be needed. The thresholds will be implemented in the real-time Surveillance and Prevention of the impacts of Extreme Meteorological Events on public health (SUPREME) system of the Institut National de Santé Publique du Québec [50] in order to alert public health stakeholders and, eventually, hospital administrators.

This study also reminds us that using internationally set thresholds may underestimate the specific dose–response relationship that exists in a specific context. The actual health impacts in a given area takes into account the local nature of the pollutants, their dispersion, the sensitivity of the local population given its demographics and health status, and their various behaviors that can augment or reduce exposure. Such adapted thresholds could also speak more to local decision-makers as they reflect the health impacts of their citizens. More studies examining the cost-benefits involved would be of interest to implement such an approach more widely [51].

## Figures and Tables

**Table 1:** Mean daily values of each variable considered in the present study. PM_2.5_ = fine particle matters; O_x_ = combined oxidant capacity of ozone and nitrogen dioxide.

Variable	Montreal		Quebec City	
	Summer	Winter	Summer	Winter
Mortality count	33.6	39.5	3.1	3.9
Max PM_2.5_ (μg/m^3^)	18.4	19.9	17.3	19.7
Max O_x_ (ppb)	28.1	22.0	24.9	23.9
Temperature (°C)	17.9	−1.0	14.4	−4.3
Relative humidity (%)	67.0	69.0	65.1	66.6

**Table 2:** Descriptive statistics of the estimated excess mortality (EM) for the period 2000–2015 in Montreal, expressed in percentage (%). Summer spans the months May–September and winter the months October–April.

	Minimum	1st Quartile	Median	Mean	3rd Quartile	Maximum	Standard Deviation
**Summer**	−52.4	−13.4	−0.7	0.1	11.6	110.4	18.3
**Winter**	−47.5	−11.2	−0.6	0.0	10.4	58.8	16.1

**Table 3:** Results for indicator weightings and thresholds for Montreal and Quebec. FA = false alarm.

City	Season	PM_2.5_ (μg/m^3^)			O_x_ (ppb)			Sensitivity (%)		FA per Year	
		$\alpha_{0}$	$\alpha_{1}$	s	$\alpha_{0}$	$\alpha_{1}$	s	Days	Episodes	Days	Episodes
Montreal	Summer	0.9	0.1	31	0.5	0.5	43	22.4	87.5	3.1	1.5
	Winter	0.5	0.5	25	0.8	0.2	26	15.4	71.4	8.0	3.7
Quebec City	Summer	0.5	0.5	32	0.8	0.2	23	20.4	85.7	4.7	2.6
	Winter	0.5	0.5	33	0.7	0.3	21	9.5	50	15.5	7.4

**Table 4:** Sample of indicators and threshold guidelines from different organizations. * estimated values of O_x_ based on the guideline values of O_3_ and NO_2_ using the formula of Section 2.1.

Geographic Scale	PM_2.5_		O_3_		NO_2_		O_x_	Reference
	Indicator	Threshold (μg/m^3^)	Indicator	Threshold (ppb)	Indicator	Threshold (ppb)	Threshold (ppb)	
World	24-h mean	25	8-h mean	50	1-h mean	106	69 *	[19]
Canada	24-h mean	27	8-h mean	62	1-h mean	60	61 *	[16]
Province of Quebec	1-h mean	30	1-h mean	80	1-h mean	213	125 *	[18]
Montreal	3-h mean	35	-	-	-	-	-	[46]

## Supplementary Materials

The following are available online at https://www.mdpi.com/1660-4601/16/12/2095/s1, Figure S1: Lag–response relationship between mortality in Quebec City and (a) PM_2.5_ and (b) O_3_. Figure S2: Number of excess mortality (EM) episodes according to the chosen preliminary EM threshold $s_{OM}$ for Quebec City. Figure S3: EM series with the identification of EM episodes for Quebec City. Figure S4: DLNM surfaces obtained between mortality and PM_2.5_ as well as O_x_ for (a, b) summer and (c, d) winter. Figure S5: Excesses of cardiovascular and respiratory (a) hospital admissions and (b) the sum of hospital admission and deaths for summer in Montreal as an example. Table S1: Best weights and threshold candidates for the summer air pollution-health warning system (APHWS) in Quebec City. Table S2: Best weights and threshold candidates for the winter APHWS in Quebec City. Table S3: Episodes with EM > 50% and PM_2.5_ max > 25 μg/m^3^ for the summer APHWS in Montreal. Table S4: Episodes with EM > 40% and PM_2.5_ max > 25 μg/m^3^ for the winter APHWS in Montreal. Table S5: Best weights and threshold candidates for the summer APHWS in Montreal. Table S6: Best weights and threshold candidates for the winter APHWS in Montreal.
