# Supplementary material for: Toward an Improved Air Pollution Warning System in Quebec
Source: Int J Environ Res Public Health. 2019 Jun 13;16(12):2095. doi: 10.3390/ijerph16122095 (PMC6617323; doi:10.3390/ijerph16122095)
Supplement: Supplementary file 1 [file ijerph-16-02095-s001.pdf]

Supplementary Materials

Toward an Improved Air Pollution Warning System in Quebec

Masselot P., Chebana F, Campagna C., Lavigne É, Ouarda T, Gosselin P.

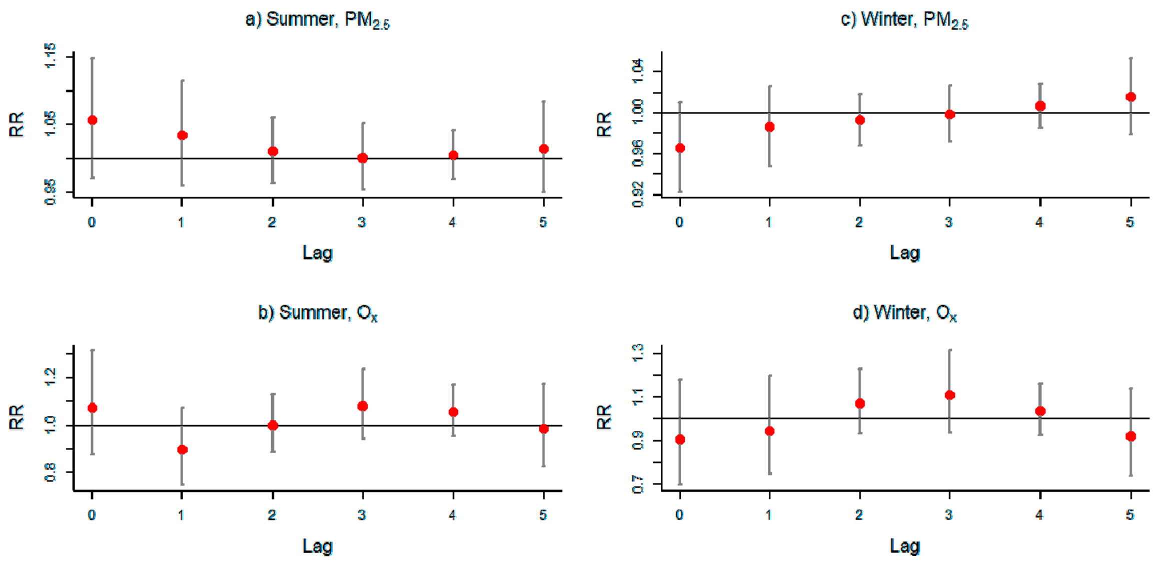

Figure S1. Lag–response relationship between mortality in Quebec and (a) PM2.5 and (b) O_3. Since no lag is significant according to the 95% confidence interval, maximum lag $\boldsymbol{L} = \mathbf{1}$ is chosen.

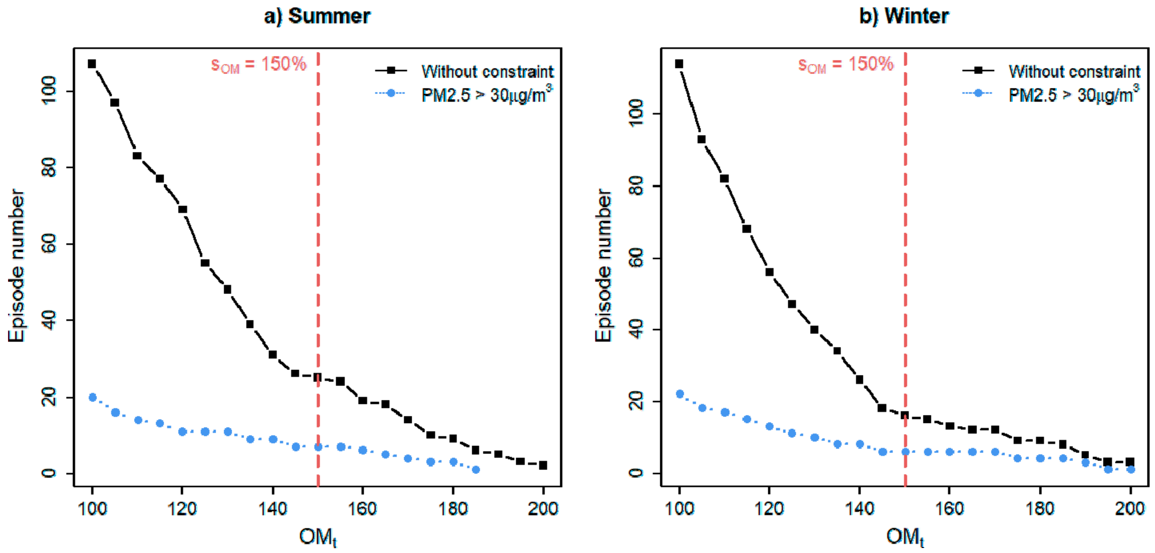

Figure S2. Number of excess mortality (EM) episodes according to the chosen preliminary EM threshold s_{OM} for Quebec. The choices of 150% correspond to breakpoints in the number of episodes.

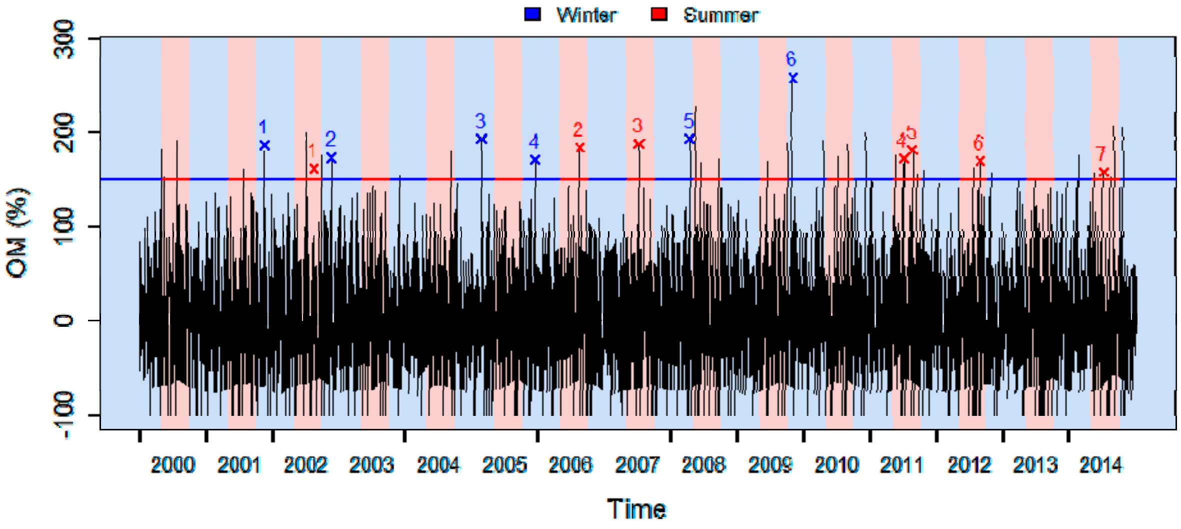

Figure S3. EM series with the identification of EM episodes for Quebec. Crosses indicate extreme EM days (exceeding s_{OM} = 150%) and the number identifies episodes. Note that non-extremes days extending the episodes are not identified here for clarity purposes. Background color separate winter (blue) and summer (red). Here, many extreme days are not linked to air pollution and the response to $PM_{2.5}$ is thus less obvious than in Montreal.

Table S1. Best weights and threshold candidates for the summer APHWS in Quebec. The chosen one is highlighted in green. FA = false alarm.

$PM_{2.5}$			O_3			Sensitivity (%)		FA per Year	
α_0	α_1	s	α_0	α_1	s	Days	Episodes	Days	Episodes
1.0	0.0	34	0.6	0.4	23	22.4	100.0	6.0	3.7
0.5	0.5	32	0.8	0.2	23	20.4	85.7	4.7	2.6
0.5	0.5	32	1.0	0.0	25	16.3	71.4	3.9	2.0
0.5	0.5	30	1.0	0.0	29	16.3	57.1	4.0	2.0
0.5	0.5	31	1.0	0.0	29	14.3	42.9	3.2	1.5

Table 2. Best weights and threshold candidates for the winter APHWS in Quebec. The chosen one is highlighted in green.

$PM_{2.5}$			O_3			Sensitivity (%)		FA per Year	
α_0	α_1	s	α_0	α_1	s	Days	Episodes	Days	Episodes
0.9	0.1	20	0.6	0.4	21	26.2	66.7	54.9	14.3
0.5	0.5	33	0.7	0.3	21	9.5	50.0	15.5	7.4
1.0	0.0	50	1.0	0.0	23	4.8	33.3	3.0	2.1

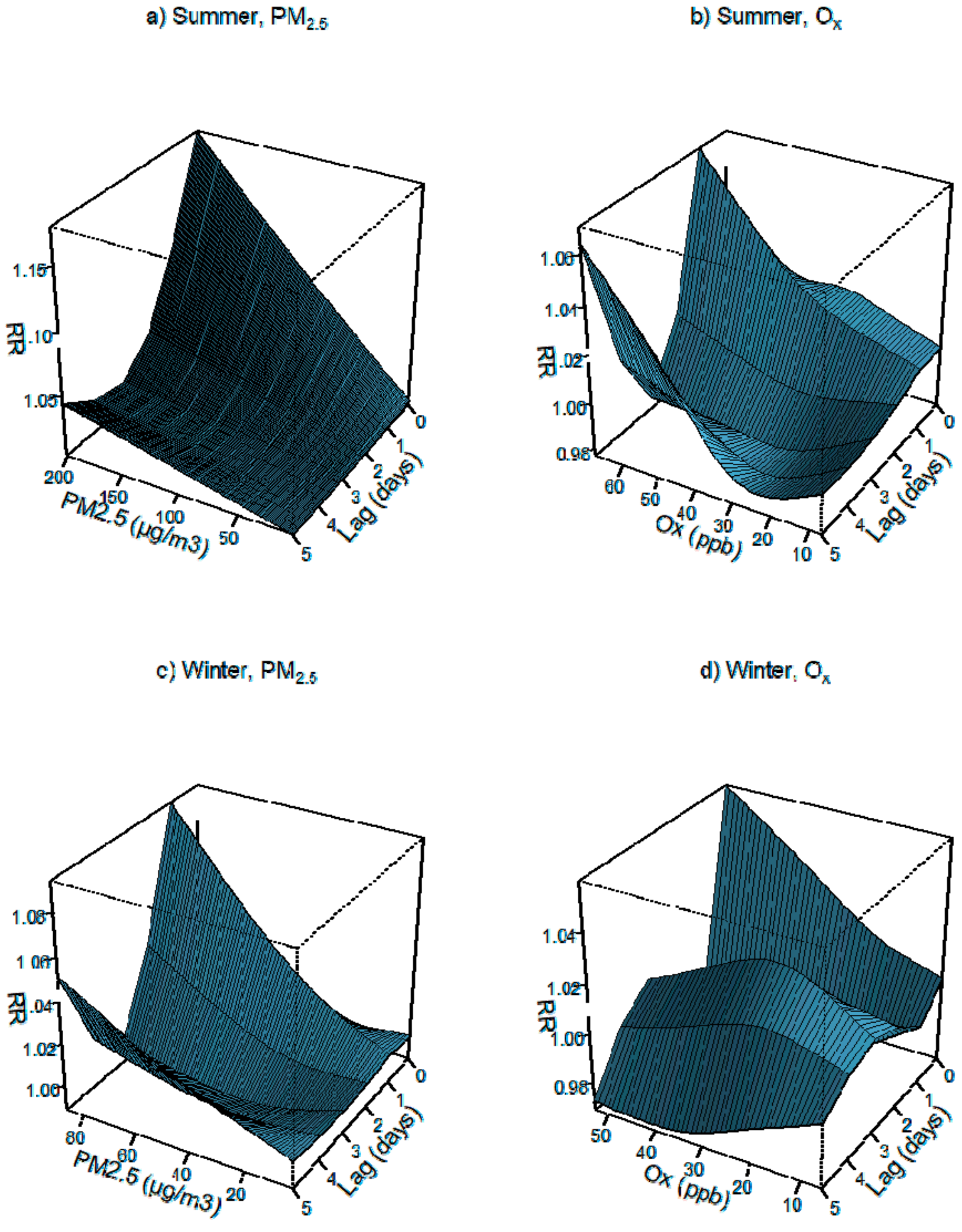

Figure S4. DLNM surfaces obtained between mortality and $PM_{2.5}$ as well as O_x for (a, b) summer and (c, d) winter. RR = Relative risk

Table S3. Episodes with EM > 50% and $PM_{2.5}$ max > 25 μg/m^3 for the summer APHWS in Montreal.

Episode	Date	EM (%)	$PM_{2.5}$ max (μg/m^3)	O_x max (ppb)	Tmean (°C)	Relative Humidity (%)
1	2 May 2001	57	35	60	21	40
2	15 June 2001	52	40	55	26	47
	16 June 2001	69	42	43	27	67
3	10 August 2001	59	36	44	25	73
4	3 July 2002	74	31	43	29	71
5	4 July 2004	51	40	28	22	44
6	13 June 2005	53	32	27	27	75
7	3 August 2007	51	32	45	26	68
8	8 July 2010	110	38	35	29	67
	9 July 2010	91	33	35	26	73

Table 4. Episodes with EM > 40% and $PM_{2.5}$ max > 25 μg/m^3 for the winter APHWS in Montreal.

Episode	Date	EM (%)	$PM_{2.5}$ max (μg/m^3)	O_x max (ppb)	Tmean (°C)	Relative Humidity (%)
1	18 March 2003	53	29	31	−0	68
	21 March 2003	52	27	19	4	76
2	4 January 2006	46	26	19	−8	61
3	10 March 2009	44	31	29	−3	61
4	3 April 2010	50	29	45	18	48
5	12 January 2013	56	27	16	2	81
6	24 February 2013	54	31	23	0	92
7	22 April 2014	43	33	26	9	84

Table 5. Best weights and threshold candidates for the summer APHWS in Montreal. The chosen one is highlighted in green.

$PM_{2.5}$			O_3			Sensitivity (%)		FA Per Year	
α_0	α_1	s	α_0	α_1	s	Days	Episodes	Days	Episodes
0.7	0.3	30	1.0	0.0	42	25.8	100.0	4.2	2.0
0.9	0.1	31	0.5	0.5	43	22.4	87.5	3.1	1.5
1.0	0.0	34	0.5	0.5	43	15.5	75.0	2.7	1.3
1.0	0.0	32	0.7	0.3	45	15.5	62.5	2.3	1.1
1.0	0.0	32	1.0	0.0	50	10.3	50.0	1.8	0.9

Table 6. Best weights and threshold candidates for the winter APHWS in Montreal. The chosen one is highlighted in green.

$PM_{2.5}$			O_3			Sensitivity (%)		FA per Year	
α_0	α_1	s	α_0	α_1	s	Days	Episodes	Days	Episodes
0.8	0.2	29	0.8	0.2	21	21.2	100.0	15.8	6.7
1.0	0.0	32	0.9	0.1	21	17.3	85.7	12.3	6.5
0.5	0.5	25	0.8	0.2	26	15.4	71.4	8.0	3.7
0.5	0.5	25	0.6	0.4	22	11.5	57.1	18.5	2.3
0.6	0.4	27	0.5	0.5	31	7.6	42.9	2.0	0.9

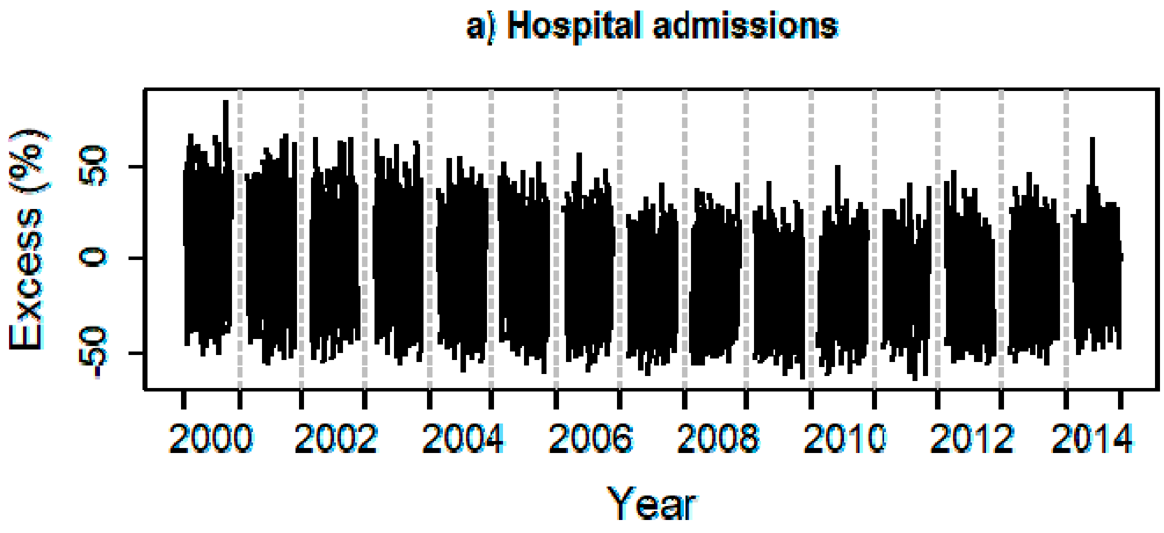

Figure S5. Excesses of cardiovascular and respiratory (**a**) hospital admissions and (**b**) the sum of hospital admission and deaths for summer in Montreal as an example. Excesses are computed as explained in Section 2.2.2. of the main manuscript.
